# The inhibitory effect of human umbilical cord mesenchymal stem cells expressing anti-HAAH scFv-sTRAIL fusion protein on glioma

**DOI:** 10.3389/fbioe.2022.997799

**Published:** 2022-11-08

**Authors:** Tian Xue, Xiaolin Wang, Jing Ru, Lixing Zhang, Huancai Yin

**Affiliations:** ^1^ College of Life Sciences, Northwest University, Xi’an, China; ^2^ Department of Plastic and Burn Surgery, The Second Affiliated Hospital, Air Force Medical University, Xi’an, China; ^3^ CAS Key Lab of Bio-Medical Diagnostics, Suzhou Institute of Biomedical Engineering and Technology, Chinese Academy of Sciences, Suzhou, China

**Keywords:** human umbilical cord mesenchyreal stem cells, single-chain antibody (scFv), human aspartyl-(asparaginyl)-β-hydroxylase, tumor necrosis factor-related apoptosis-inducing ligand (TRAIL), glioma

## Abstract

Glioma is the most common malignant intracranial tumor with low 5-year survival rate. In this study, we constructed a plasmid expressing anti-HAAH single-chain antibody and sTRAIL fusion protein (scFv-sTRAIL), and explored the effects of the double gene modified human umbilical cord mesenchyreal stem cells (hucMSCs) on the growth of glioma *in vitro* and *in vivo*. The isolated hucMSCs were identified by detecting the adipogenic differentiation ability and the osteogenic differentiation ability. The phenotypes of hucMSCs were determined by the flow cytometry. The hucMSCs were infected with lentivirus expression scFv-sTRAIL fusion protein. The expression of sTRAIL in hucMSCs were detected by immunofluorescence staining, western blot and ELISA. The tropism of hucMSCs toward U87G cells was assessed by transwell assay. The inhibitory effect of hucMSCs on U87G cells were explored by CCK8 and apoptosis assay. The xenograft tumor was established by subcutaneously injection of U87G cells into the back of mice. The hucMSCs were injected *via* tail veins. The inhibitory effect of hucMSCs on glioma *in vivo* was assessed by TUNEL assay. The hucMSCs migrated into the xenograft tumor were revealed by detecting the green fluorescent. The results showed that the scFv-sTRAIL expression did not affect the phenotypes of hucMSCs. The scFv-sTRAIL expression promoted the tropism of hucMSCs toward U87G cells, enhanced the inhibitory effect and tumor killing effect of hucMSCs on U87G cells. The *in vivo* study showed that hucMSCs expressing scFv-sTRAIL demonstrated significantly higher inhibitory effect and tumor killing effect than hucMSCs expressing sTRAIL. The green fluorescence intensity in the mice injected with hucMSCs expressing scFv-sTRAIL was significantly higher than that injected with hucMSCs expressing sTRAIL. These data suggested that the scFv conferred the targeting effect of hucMSCs tropism towards the xenograft tumor. In conclusion, the hucMSCs expressing scFv-sTRAIL fusion protein gained the capability to target and kill gliomas cells *in vitro* and *in vivo*. These findings shed light on a potential therapy for glioma treatment.

## Introduction

Glioma is the most common primary intracranial tumor, and it is derived from neuroepithelium and accounts for 50%–60% of brain tumors (Grady et al., 2022; O'Connor et al., 2021). At present, the clinical treatment of glioma mainly includes neurosurgical resection, radiotherapy, chemotherapy, and immunotherapy (Grady et al., 2022). Nevertheless, patients have a mere median survival of 14 months, as well as a 5-year survival rate less than 10% (Birk et al., 2017). The recurrence rate of high-grade gliomas (WHO grade III-IV) is as high as 95% within 2 years after initial resection (Xiong et al., 2019). Drug or gene delivery vehicles commonly used to treat cancers, such as glioma include liposomes, nanoparticles, ionic polymers, microcapsules, and micropores (Ren et al., 2021). However, the clinical therapeutic value of these vectors is limited by factors, such as low drug or gene load, poor stability, and low targeting efficiency (Vikulina et al., 2019; Stephen et al., 2022). Therefore, cell-based targeted drug (gene) delivery systems have gradually attracted worldwide attention due to their unique advantages.

Cells that can be used as drug or gene delivery vehicles include red blood cells, immune cells, and stem cells (Wu et al., 2019). As a new type of drug or gene delivery cell carrier, human umbilical cord mesenchyreal stem cells (hucMSCs) present unique characteristics (Qu et al., 2020). The hucMSCs exhibit a faster renewal rate, stronger proliferation potential, and stronger immunosuppressive ability compared to adult mesenchymal stem cells derived from other mature tissues (Xu et al., 2018; Zha et al., 2021). Harvesting the umbilical cord cells is non-invasive and painless and has a wide range of sources without any ethical controversy (Coccini et al., 2022). Moreover, studies have reported that hucMSCs have the potential to cross the blood–brain barrier (BBB) (Sun et al., 2018).

Human aspartyl-(asparaginyl)-β-hydroxylase (HAAH), also known as spartate β-hydroxylase (ASPH), is a type II transmembrane protein and member of the alpha-ketoglutarate-dependent dioxygenase family (Lin et al., 2019). Its function is to hydroxylate aspartyl and asparagine residues in the epidermal growth factor-like domain of the synthesized protein (Greve et al., 2021). HAAH is not expressed in normal tissues, but highly expressed in a variety of tumor cells, including glioma (Yang et al., 2010; Chen et al., 2019; Lin et al., 2019), and is regarded as a broad-spectrum tumor-associated antigen (Zheng et al., 2020). It is a membrane protein expressed on the surface of tumor cells, and can be used for the accurate localization of cancer lesions (Babich et al., 2022).

Tumor necrosis factor-related apoptosis-inducing ligand (TRAIL) is a member of the TNF superfamily of cytokines (Burgaletto et al., 2020). TRAIL can induce apoptosis of tumor cells but not normal cells (Yuan et al., 2018). TRAIL can specifically induce apoptosis by selectively binding to the TRAIL receptors on the surface of cancer cells, such as colon cancer cells, lung cancer cells, and glioma cells (Ion et al., 2019; Yoon et al., 2021). Under the MSCs homing effect, TRAIL-MSCs exhibit a tendency for homing to glioma lesions, significantly improving the local concentration of TRAIL in glioma lesions (Park et al., 2021). TRAIL can exist on the cell surface in the form of membrane protein (Wong et al., 2019). It can also be hydrolyzed by protease, shed from the cell membrane, and free in body fluids or blood in the form of soluble protein (sTRAIL) (Koliaki and Katsilambros, 2022). Studies have shown that the sTRAIL, like the membrane-bound form TRAIL, can induce apoptosis in a variety of tumor cell lines without affecting normal cells (Ma et al., 2005; de Bruyn et al., 2013).

A single-chain antibody (scFv) is a widely used recombinant antibody (Eskafi et al., 2021). It is a smaller unit with the ability to bind antigen. The advantages of scFv include lower molecular weight and easy modification (Eskafi et al., 2021). Thus, anti-HAAH scFv has potential application value in targeted therapy for glioma (Schaller et al., 2020). Currently, the use of anti-HAAH scFv for glioma-targeted therapy has not been reported yet. In this study, we constructed a plasmid expressing anti-HAAH single-chain antibody and sTRAIL fusion protein (scFv-sTRAIL), and explored the effects of the double gene modified hucMSCs on the growth of glioma *in vitro* and *in vivo*.

## Materials and methods

### Cell culture

Human glioblastoma cells U87G (kindly gifted by Prof. Hai Zhang of the Air Force Medical University) were maintained in Dulbecco’s modified Eagle’s medium (DMEM, Sigma, St. Louis, MO, United States) with 10% fetal bovine serum (FBS, Sigma) and incubated at 37°C in a humidified atmosphere of 5% CO_2_.

Human umbilical cord tissues were obtained from the Second Affiliated Hospital, Air Force Medical University. Informed consent was obtained from participants, and this study has been approved by the ethics committee of the Second Affiliated Hospital, Air Force Medical University (TDLL 2019-11-179). The hucMSCs were isolated from the Wharton’s jelly of the human umbilical cord. The Wharton’s jelly was separated in the laboratory ultra-clean workbench. After repeated washing with phosphate buffer saline (PBS), the Wharton’s jelly was cut into 1 mm^3^ tissue pieces, evenly spread in a 60 mm Petri dish, and maintained in DMEM supplemented with 10% FBS, 1% penicillin, and 1% streptomycin in an atmosphere of 37°C, 5% CO_2_. After the cells were confluent, they were digested and passaged with 0.25% trypsin. For this experiment, the hucMSCs were limited to 4th ∼ 5th generations (P4 ∼ P5). The morphology of the hucMSCs was assessed using an inverted fluorescence microscope (Niko, Tokyo, Japan).

### Identification of hucMSCs biomarkers by flow cytometry

The P4 hucMSCs were digested and PBS was added to prepare cell suspension. Cells were labeled using HLA-DR-PE, CD105-APC, CD90-PE, CD73-APC, CD19-APC, CD45-PE-Cy7, CD34-PE-Cy7 or CD14-PE antibodies (BioGems, Westlake Village, CA,United States) for 30 min at 4°C. Subsequently, cells were washed with PBS for three times. The above stained cells were analyzed using flow cytometry (BD Biosciences, San Jose, CA).

### Identification of hucMSCs differentiation by alizarin red S staining and oil red O staining

The cell suspension of P4 hucMSCs (1× 10^8^/L) was plated in six well plate with DMEM medium containing 10% FBS. When the cells confluency reached 70%, the osteoinductive complete culture medium or adipogenic differentiation complete culture medium (Guangzhou Cyagen biotechnology Co., Ltd., Guangzhou, China) was added, which was changed every 3 days. After 21 days, cells were stained using 0.1% alizarin red S (Sigma) or 2% oil red O (Cyagen biotechnology Co., Ltd.). The formation of calcified nodules or lipid droplet were observed under microscope.

### Construction of plasmids expressing anti-HAAH scFv-sTRAIL fusion protein

**FIGURE 1:**
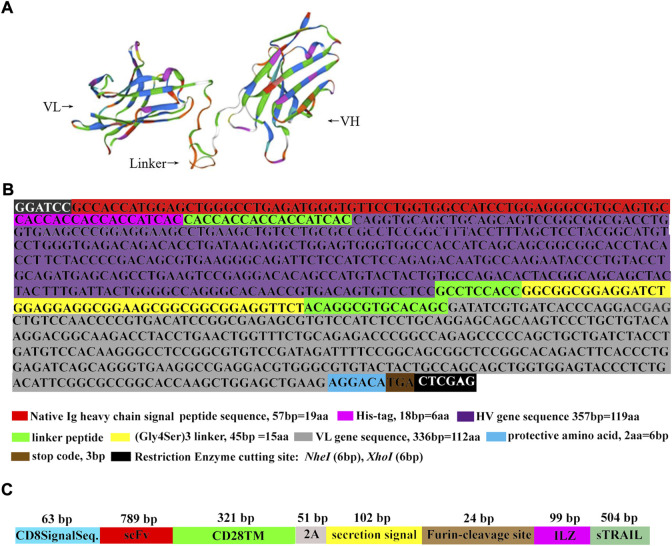
The schematic diagram of scFv and scFv-sTRAIL. **(A)** The mimic tertiary structure of scFv protein. **(B)** The results of scFv sequence determination. **(C)** The gene linear structural pattern diagram of the sequence encoding scFv-sTRAIL fusion protein.

The hybridoma cell line G3/F11 secreting anti-HAAH monoclonal antibody (McAb) was donated by Prof. Yingfeng Lei from the Microbiology Department of Air Force Military Medical University (Yang et al., 2011). The plasmids expressing anti-HAAH scFv were constructed according to previously described (Yang et al., 2011). Briefly, the total RNA of G3/F11 was extracted using RNeasy Mini Kit (Qiagen, Valencia, CA) according to the instruction. The cDNA was synthesized *via* Transcriptor First Strand cDNA Synthesis Kit (Roche, Maryland, United States). Genes encoding the variable heavy and light chains (VH and VL) of the anti-HAAH antibodies were amplificated by polymerase chain reaction (PCR) using Taq DNA polymerase (Thermo Fisher Scientific, MA, United States) in a thermocycler (QIAGEN GmbH, Hilden, Germany). Based on the correctly sequenced VH and VL gene sequences, we designed the upstream and downstream restriction endonuclease sites (*BamHI* and *XhoI*), signal peptide, His-tag, Linker and other elements. The mimic tertiary structure of scFv protein was showed by SWISS-MODEL (Figure 1A). Based on the designed scFv gene sequence (Figure 1B) and the sTRAIL gene sequence at NCBI, the sequence encoding scFv-sTRAIL was designed. The gene linear structural pattern diagram encoding the scFv-sTRAIL fusion protein in this study was shown in Figure 1C. The sequences encoding scFv-sTRAIL fusion protein were chemically synthesized, cloned into pCDH-CMV-MSC-EF1-copGFP-T2A-Puro plasmids, and packaged into a lentivirus by Hanheng Biotech (Shanghai, China).

### Infection of hucMSCs with lentivirus

The cell suspension of P4 hucMSCs was plated in six well plate. When cells were 70% confluent, hucMSCs were infected with lentivirus expressing empty plasmids (LV-NC group), lentivirus expressing sTRAIL (LV-sTRAIL group), or lentivirus expressing scFv-sTRAIL (LV-scFv-sTRAIL group). Medium containing puromycin (10 μg/ml; Invitrogen) was used to select stably infected cells. The hucMSCs normally cultured without infection were defined as control group.

### Immunofluorescence staining

The hucMSCs were seeded into a 6-well dish containing cover slips and incubated for 48 h. The cells were taken out and were then washed twice using PBS, fixed for 15 min with 95% ethanol, permeabilized in 0.1% Triton X-100 and blocked for 1 h using 1% BSA in PBST. Cells were then incubated with mouse anti-TRAIL antibodies (ProteinTech Group, Wuhan, China) at 4°C overnight. After that, slides were washed three times with PBS. Then cells were incubation with Cy3-conjugated affinipure goat anti-mouse IgG (ProteinTech Group, Wuhan, China) and DAPI (1 μg/ml; Thermo Fisher Scientific, Inc.) in the dark for 1 h at room temperature. After washing with TBS and distilled water, fluorescent images were collected using Olympus FV3000RS confocal microscope, and fluorescent signal was quantified using ImageJ.

### Enzyme linked immunosorbent assay

ELISA was performed to detect the concentration of sTRAIL in culture supernatant of hucMSCs using the sTRAIL ELISA kit (Diaclone Research, Besancon, France). Absorbance was measured at 450 nM using a microplate reader (Bio-Rad Laboratories, Inc.). All samples were analyzed in triplicate.

### Western blot

Western blot was used to verify sTRAIL protein expression in hucMSCs. Cells was lysed with RIPA buffer (Millipore, Bedford, MA. United States), and the protein concentration was tested by a Bradford protein assay kit (TIANGEN Co., Ltd., No.PH0325). Protein samples were separated by SDS-PAGE and transferred onto polyvinylidene fluoride membrane (Millipore). After blocking them with 3% nonfat milk, the membranes were incubated with rabbit anti-TRAIL antibody (Beyotime, Nantong, China) (1:200) and HRP-goat anti-rabbit IgG (CST) (1:5,000) successively. The proteins were detected by enhanced chemiluminescence. Finally, the bands were visualized using a Bio-Rad ChemiDoc apparatus (Bio-Rad, Hercules, CA, United States) and were analyzed using ImageJ software (ImageJ, Version 1.4). GAPDH was used as the internal reference.

### Migration assay

Transwell migration assay was used to monitor the tropism of the hucMSCs towards glioma U87G cells. For this purpose, U87G cells (1 × 10^5^/well) were plated in the lower chamber of the transwell plate (Thermo Fisher Scientific, Waltham, MA, United States). The hucMSCs (1 × 10^4^/well) in each group were grown in the upper chamber. Cells in each group were incubated for 24 h at 37°C with 5% CO_2_. The non-migrating cells in the upper chamber were gently wiped away with a cotton swab. The bottom of the chamber was stained with methanol and 0.1% crystal violet and then observed using a microscope. The number of invaded hucMSCs per field was hucMSCs number at an average of five-random non-overlapping fields.

### Detection of U87G proliferation by CCK8 assay

U87G cells were seeded in 96-well plates (5 × 10^4^/well) and pre-cultured for 24 h at 37°C with 5% CO_2_. Subsequently, the culture supernatant concentrate (1:10, 100 μl/well) of hucMSCs in each group was added, followed by incubation for 24 h. A total of 10 μl/well of CCK8 reagent (Merck KGaA, Darmstadt, Germany) was added, followed by further incubation for 2–4 h. The absorbances were measured at OD450 nm using a microplate reader (Bio-Rad Laboratories, CA, United States).

### Measurement of U87G apoptosis by flow cytometry

In this study, the effect of hucMSCs infected with LV-scFv-sTRAIL on U87G apoptosis was investigated using co-culture assay. For co-culture, U87G cells (1 × 10^5^/well) were plated in the lower chamber of the transwell plate (Thermo Fisher Scientific) and incubated for 24 h. Then, hucMSCs (1 × 10^4^/well) in each group were grown in the upper chamber. After another 48 h, glioma cell apoptosis was detected using the Annexin V-APC/PI apoptosis detection kit (Invitrogen, Carlsbad, CA, United States). Briefly, the cells were resuspended in 300 µl binding buffer, followed by the addition of Annexin V-APC solution (5 µl). After 25 min of incubation at 4°C, the cells were resuspended for 10 min in a binding buffer with 5 μl of PI. Apoptotic cells were counted using a FACS analyzer (BD Biosciences).

### Xenograft tumor assay

The immunodeficient BGR mice (SPF grade, 6–8 weeks old, 18–20 g) were purchased from the Laboratory Animal Center of Air Force Military Medical University. The experiments were carried out in strict accordance with the guidelines of the National Health and Medical Research Council for the Care and Use of Animals for Experimental Purposes in China, and were approved by the Institutional Animal Care and Use Committee of Chinese Academy of Sciences (No. 2020-A138). Glioma U87Gcells (2 × 10^6^ cells) diluted in 200 µl of PBS were inoculated subcutaneously into the back of BGR mice. The tumor volume was calculated every 2 days by a vernier caliper: The volume was calculated as follows: volume = length × width^2^ × 1/2. When the tumor volume reached 10–20 mm^3^, mice were randomly divided into four groups (five mice/group) as follows: hucMSCs group (injected with control hucMSCs), hucMSCs-LV-NC group (injected with hucMSCs infected with pCDH empty plasmid lentivirus), hucMSCs-LV-sTRAIL group (injected with hucMSCs infected with LV-sTRAIL), and hucMSCs-LV-scFv-sTRAIL group (injected with hucMSCs infected with LV-scFv-sTRAIL). The mice in each group were injected with 1 × 10^7^ cells (in 200 μl suspension) *via* their tail veins once a week for a total of four times. One week after the last inoculation, the mice were euthanized by subcutaneous injection with sodium pentobarbital (40 mg/kg). The tumors were isolated and the tumors weight were measured.

Because the lentiviral plasmids pCDH-CMV-MSC-EF1-copGFP-T2A-Puro used in this study could express GFP, hucMSCs migrated into the xenograft tumor could be reflected by detecting the green fluorescent. Briefly, tumor tissue were paraffin-embedded and 5 μm sections were made. Green fluorescent were visulized using Olympus FV3000RS confocal microscope, and fluorescent signal was quantified using ImageJ.

### TUNEL assay

The cell apoptosis in xenograft tumors were detected by the one-step TUNEL cell apoptosis detection kit (Beyotime, Nantong, China). Briefly, the slices were blocked with anti-fluorescence quenching blocking solution and then observed under a fluorescent microscope. The excitation wavelength of Cy3 is 550 nm, and the emission wavelength is 570 nm (red fluorescence). Fluorescent images were collected using Olympus FV3000RS confocal microscope, and fluorescent signal was quantified using ImageJ. The apoptosis rate = (the mean number of apoptotic cells in five random fields/total cell count in that five fields) ×100%.

### Assessment of distribution of double gene modified hucMSCs *in vivo* by RT-PCR

The immunodeficient BGR mice (SPF grade, 6–8 weeks old, 18–20 g) were injected with LV-scFv-sTRAIL infected hucMSCs (5 × 10^7^ cells in 200 μl suspension) *via* tail veins. At the 24, 48, 72 and 96 h, the mice were euthanized by subcutaneous injection with sodium pentobarbital (40 mg/kg). The brain, spleen, liver, kidney, heart and lung were collected for total RNA extraction. The cDNA was synthesized *via* Transcriptor First Strand cDNA Synthesis Kit (Roche). The presence of double gene modified hucMSCs were revealed by RT-PCR analysis for GFP (Cafforio et al., 2017), and β-actin was used as loading control. The primer sequences used were as follows: GFP: 5′-AGG​ACA​GCG​TGA​TCT​TCA​CC-3’ (sense) and 5′-CTT​GAA​GTG​CAT​GTG​GCT​GT-3’ (antisense); and β-actin: 5′-ACA​GAG​CCT​CGC​CTT​TGC-3’ (sense), 5′-GCG​GCG​ATA​TCA​TCA​TCC-3’ (antisense).

### Statistical analysis

Data were presented as means ± standard deviation (SD) of three independent experiments. For multiple-group comparisons, a significant one-factor analysis of variance (ANOVA) was used, followed by the Bonferroni test. *p* < 0.05 was considered to indicate a statistically significant difference. Analyses were performed using GraphPad Prism 5 (GraphPad Software Inc.; San Diego, CA, United States).

## Results

### Identification of the isolated hucMSCs

**FIGURE 2:**
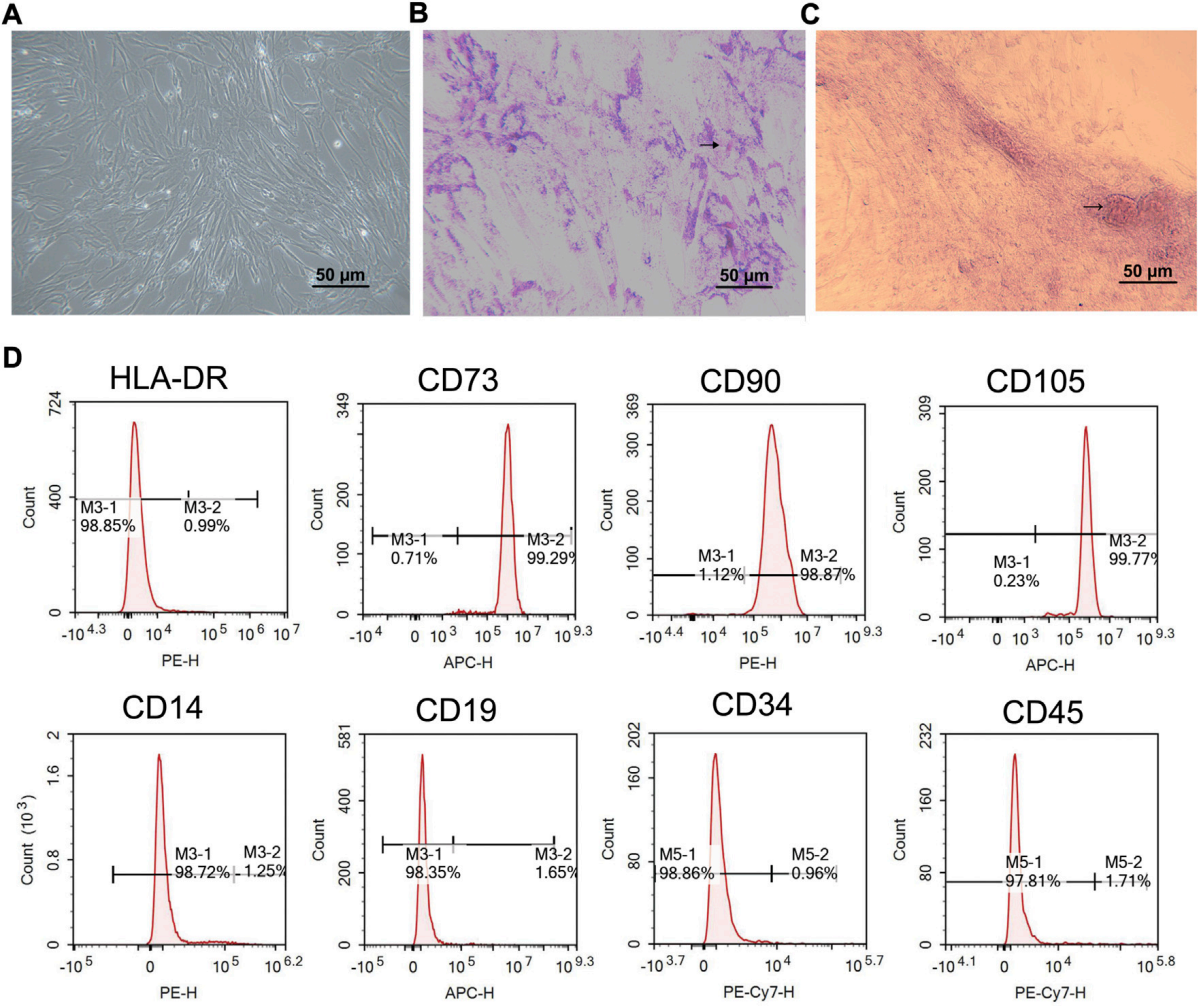
Characterization of hucMSCs. **(A)** Phase contrast picture of confluent huMSCs (×100). **(B)** Adipogenic differentiation observed with Oil Red O staining (×100). **(C)** Osteogenic differentiation observed with alizarin staining (× 100). **(D)** Flow cytometric analysis of hucMSCs specific biomarkers. Scale bar = 50 μm *n* = 3.

The hucMSCs from Wharton’s jelly of the human umbilical cord were incubated in DMEM with 10% FBS for 14 days. Spindle-forming fiber-like hucMSCs were densely arranged in a fingerprint or vortex shape (Figure 2A). After 3 weeks of adipogenic induction using the P4 cells, the formation of a large number of lipid droplets suggested that the cells have differentiated into adipocytes (Figure 2B). After osteogenic induction, the red-stained calcium nodules and scattered bone trabecular structures in the cells demonstrated the successful differentiation of cells toward osteocytes (Figure 2C). The flow cytometry results showed that CD73, CD90, and CD105 were presented as surface antigens, which are typical surface antigens of hucMSCs (Figure 2D). But the cells were CD14, CD19, CD34, CD45, and HLA-DR negative (Figure 2D). These data suggested the hucMSCs had been successfully obtained.

### The LV-scFv-sTRAIL infection did not affect the phenotypes of hucMSCs

**FIGURE 3:**
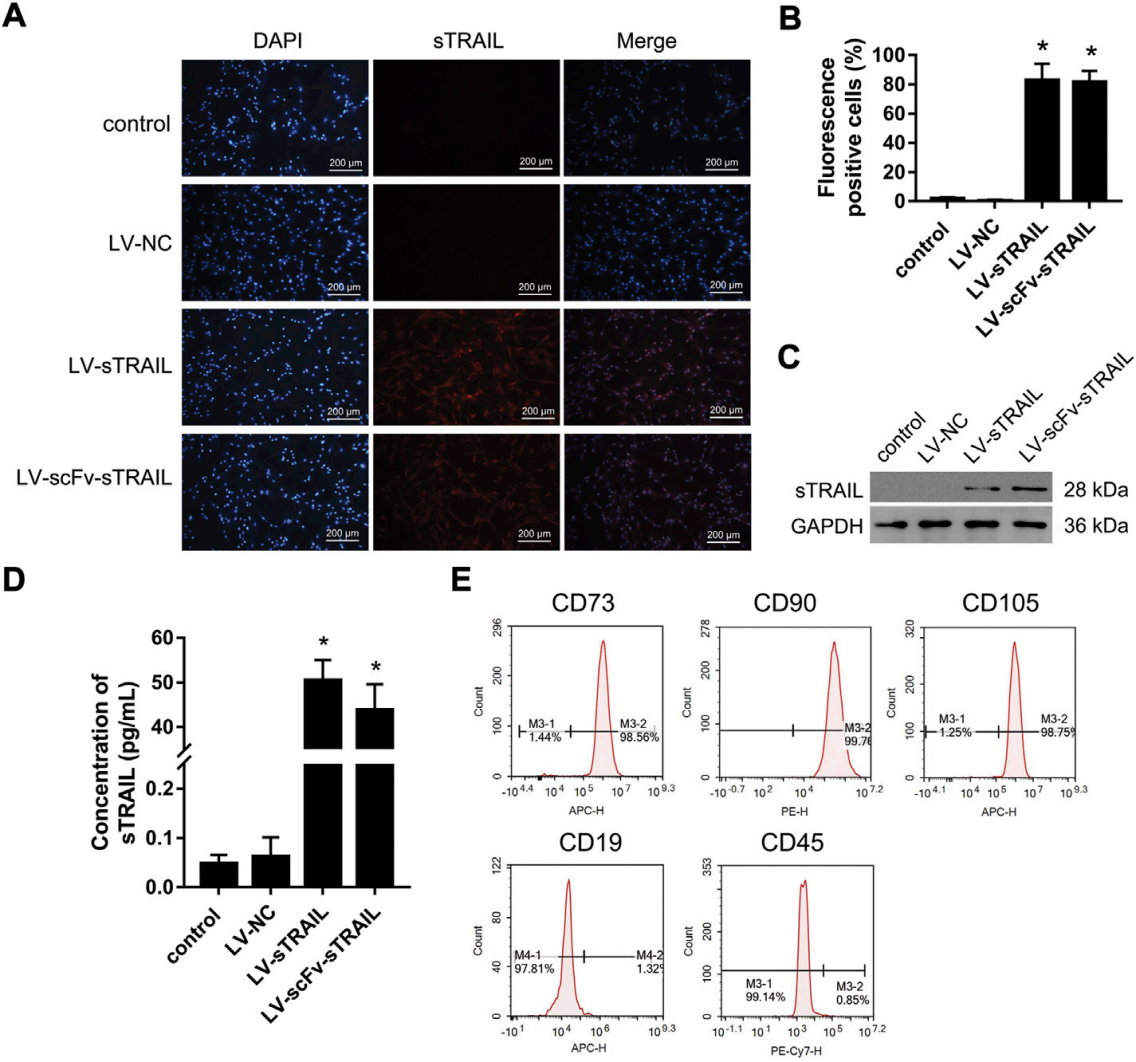
The effects of LV-scFv-sTRAIL infection on sTRAIL expression and biomarkers of hucMSCs. **(A)** The representative images of sTRAIL expression detected by immunofluorescence staining. **(B)** The quantitative data of sTRAIL positive cells. **(C)** Western blot detection of the expression of sTRAIL. **(D)** ELISA detection of the content of sTRAIL in the culture supernatant. **(E)** Flow cytometric analysis of hucMSCs specific biomarkers. Control group: normally cultured hucMSCs; LV-NC group: hucMSCs infected with empty plasmid lentivirus; LV-sTRAIL group: hucMSCs infected with lentivirus expressing sTRAIL; LV-scFv-sTRAIL group: hucMSCs infected with lentivirus expressing scFv-sTRAIL. Scale bar = 200 μm *n* = 3, *p < 0.01 versus the LV-NC group.

The hucMSCs infected with LV-sTRAIL and LV-scFv-sTRAIL both expressed sTRAIL successfully, as shown by immunofluorescence staining (Figures 3A,B) and western blot (Figure 3C). ELISA showed that the contents of sTRAIL in the culture supernatant of the LV-sTRAIL group and LV-scFv-sTRAIL group were both significantly higher than the LV-NC group (Figure 3D). There was no significant difference of the levels of sTRAIL between the LV-sTRAIL group and LV-scFv-sTRAIL group (Figures 3A–D).

Flow cytometry analysis demonstrated the hucMSCs infected with LV-scFv-sTRAIL were still CD73, CD90 and CD105 positive, and CD19 and CD45 negative (Figure 3E). This demonstrated that the LV-scFv-sTRAIL infection had no obvious effect on the phenotypes of hucMSCs.

### The effect of LV-scFv-sTRAIL infection on hucMSCs tropism towards U87G

**FIGURE 4:**
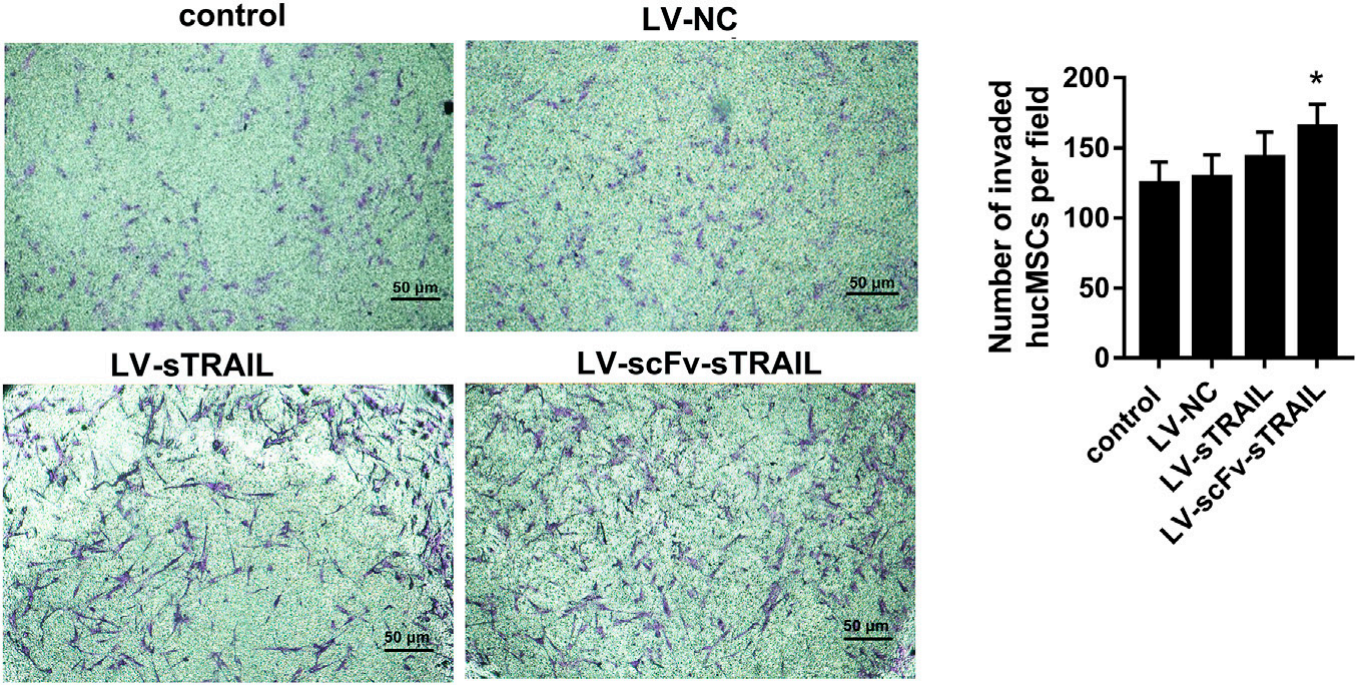
The effect of LV-scFv-sTRAIL infection on hucMSCs tropism toward U87G detected by the transwell assay (× 100). Scale bar = 50 μm *n* = 3, *p < 0.05 versus the LV-NC group.

The tropism of the hucMSCs infected with LV-scFv-sTRAIL towards U87G *in vitro* was monitored by transwell migration assay. After crystalline violet staining, the number of cells that migrated across the submembrane was counted under the microscope. The results showed that the number of transmembrane cells in the LV-scFv-sTRAIL group was significantly higher than that in the LV-NC group (Figure 4).

### The effect of hucMSCs infected with LV-scFv-sTRAIL on proliferation and apoptosis of U87G

**FIGURE 5:**
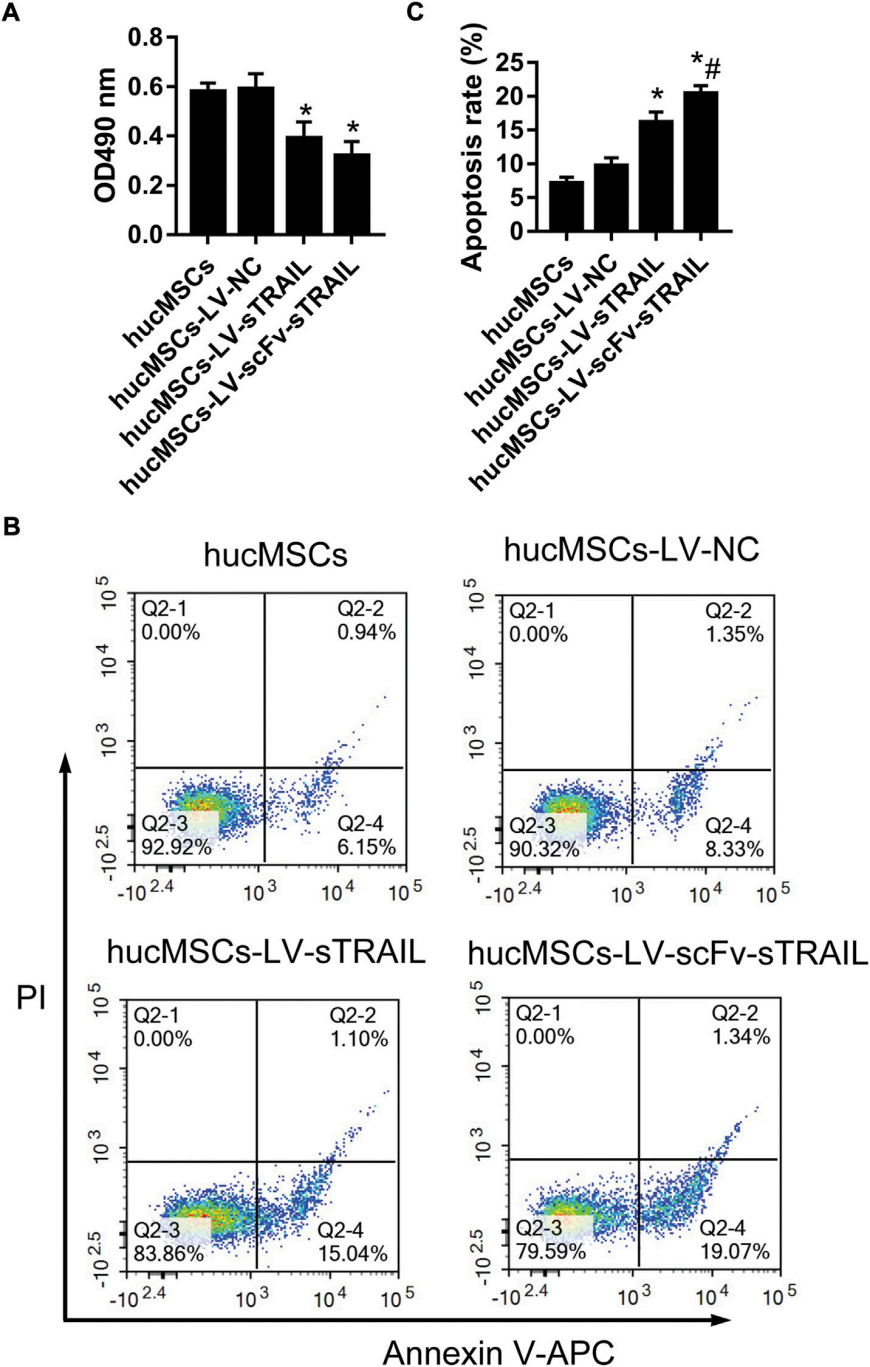
The effect of hucMSCs infected with LV-scFv-sTRAIL on proliferation and apoptosis of U87G. **(A)** The proliferation of U87G measured by CCK-8 assay. **(B)** The apoptosis of U87G measured by flow cytometric analysis. **(C)** The quantitative data of cell apoptosis. *n* = 3, *p < 0.05 versus the hucMSCs-LV-NC group; #*p* < 0.05 versus the hucMSCs-LV-sTRAIL group.

The proliferation of U87G cultured with the culture supernatant of hucMSCs in each group were measured by CCK-8 assay. The data showed that proliferation of U87G in the hucMSCs-LV-sTRAIL group and hucMSCs-LV-scFv-sTRAIL group was significantly decreased contrast to that in the hucMSCs-LV-NC group (Figure 5A). After co-culture of hucMSCs and U87G, the effect of hucMSCs on U87G apoptosis was investigated. The data revealed that the apoptosis of U87G in the hucMSCs-LV-scFv-sTRAIL group increased significantly compared to the hucMSCs-LV-NC group and hucMSCs-LV-sTRAIL group (Figures 5B,C).

### The double gene modified hucMSCs inhibited glioma tumor growth *in vivo*

**FIGURE 6:**
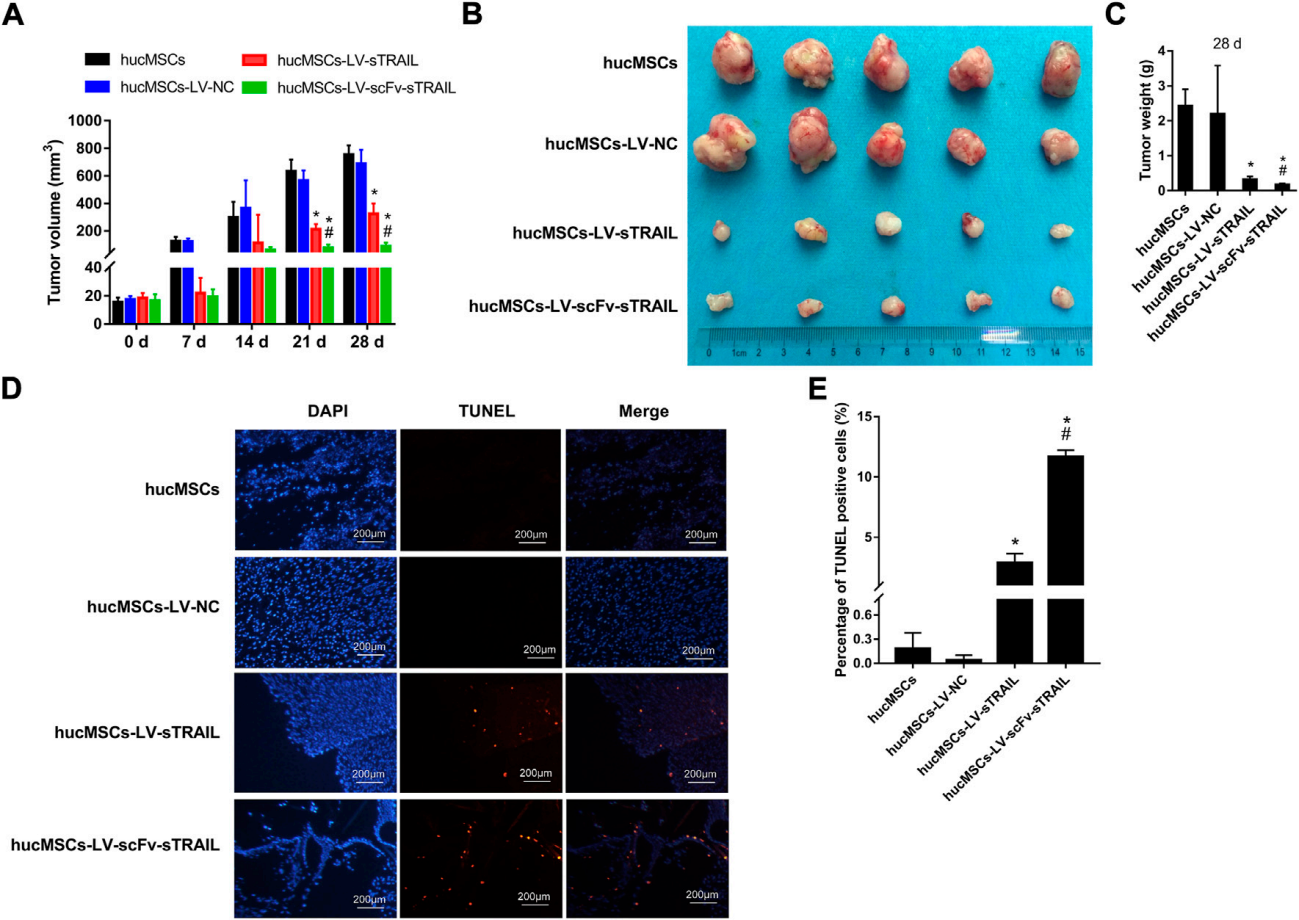
The effect of hucMSCs infected with LV-scFv-sTRAIL on glioma tumor growth. **(A)** Tumors volume. **(B)** The tumors isolated from mice. **(C)** Tumors weight on 28 days. **(D)** The representative images of apoptotic cells in U87G xenograft tumors detected by TUNEL assay. **(E)** The quantitative data of percentage of TUNEL positive cells. Scale bar = 200 μm *n* = 5, *p < 0.05 versus the hucMSCs-LV-NC group; #*p* < 0.05 versus the hucMSCs-LV-sTRAIL group.

To evaluate the effect of hucMSCs infected with LV-scFv-sTRAIL on tumor growth *in vivo*, mice with xenograft tumor were established by subcutaneous inoculation with U87G cells. The hucMSCs infected with corresponding lentivirus plasmids were injected *via* the tail veins after the xenograft tumors were established. On the 21 days and 28 days after hucMSCs injection, the tumor volume in the hucMSCs-LV-sTRAIL group and hucMSCs-LV-scFv-sTRAIL group were both significantly smaller than that in the hucMSCs-LV-NC group (*p* < 0.05, Figure 6A). 28 days after hucMSCs injection, the tumors were isolated. The data showed that the tumor weight of the hucMSCs-LV-sTRAIL group and hucMSCs-LV-scFv-sTRAIL group were both significantly smaller than that of the hucMSCs-LV-NC group (*p* < 0.05, Figures 6B,C). TUNEL assay demonstrated that the cell apoptosis rates in the U87G xenograft tumor in the hucMSCs-LV-sTRAIL group and hucMSCs-LV-scFv-sTRAIL group were both significantly up-regulated than that in the hucMSCs-LV-NC group (*p* < 0.05, Figures 6D,E). As expected, hucMSCs infected with LV-scFv-sTRAIL showed a stronger inhibitory effect on tumor growth than hucMSCs infected with LV-sTRAIL (*p* < 0.05, Figures 6A–C). The cell apoptosis rate in the hucMSCs-LV-scFv-sTRAIL group was also significantly higher than that in the hucMSCs-LV-sTRAIL group (*p* < 0.05, Figures 6D,E).

### The scFv conferred the targeting effect of hucMSCs to tumor tropism

**FIGURE 7:**
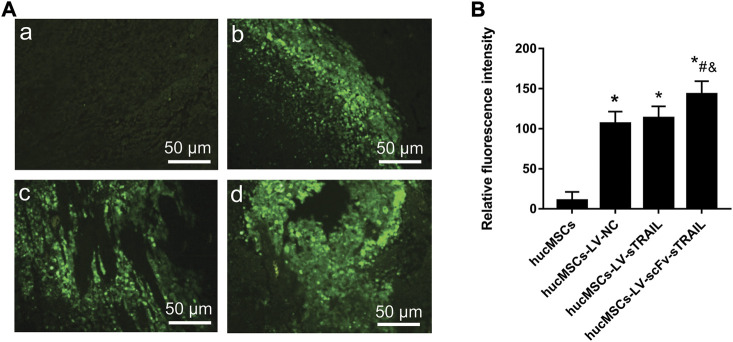
Fluorescence microscopic observation of paraffin embedded sections of tumor tissues. **(A)** Representative images of fluorescence microscope observations (magnification ×200). a: hucMSCs group; b: hucMSCs-LV-NC group; c: hucMSCs-LV-sTRAIL group; d: hucMSCs-LV-scFv-sTRAIL group. **(B)** The quantitative data of fluorescence intensity. Scale bar = 50 μm *n* = 5, *p < 0.05 versus the hucMSCs group; #*p* < 0.05 versus the hucMSCs-LV-NC group; &*p* < 0.05 versus the hucMSCs-LV-sTRAIL group.

To investigate whether the stronger inhibitory effect of hucMSCs infected with LV-scFv-sTRAIL on tumor growth was due to the targeting effect of scFv, we detected the hucMSCs migrated into the xenograft tumor. Because the lentiviral plasmids pCDH-CMV-MSC-EF1-copGFP-T2A-Puro used in this study could express green fluorescent protein (GFP), the presence of hucMSCs in the xenograft tumor could be revealed by detecting the green fluorescent. As shown in Figure 7, there was no significant difference between hucMSCs-LV-NC group and hucMSCs-LV-sTRAIL group (*p* > 0.05), suggesting the hucMSCs carrying foreign genes still maintained the tumor tropism ability *in vivo*. However, the fluorescence intensity in the hucMSCs-LV-scFv-sTRAIL group was significantly higher than that in the hucMSCs-LV-NC group and hucMSCs-LV-sTRAIL group (*p* < 0.05, Figure 7). These data suggested that the scFv conferred the targeting effect of hucMSCs tropism towards the xenograft tumor.

### Distribution of double gene modified hucMSCs *in vivo*

**FIGURE 8:**
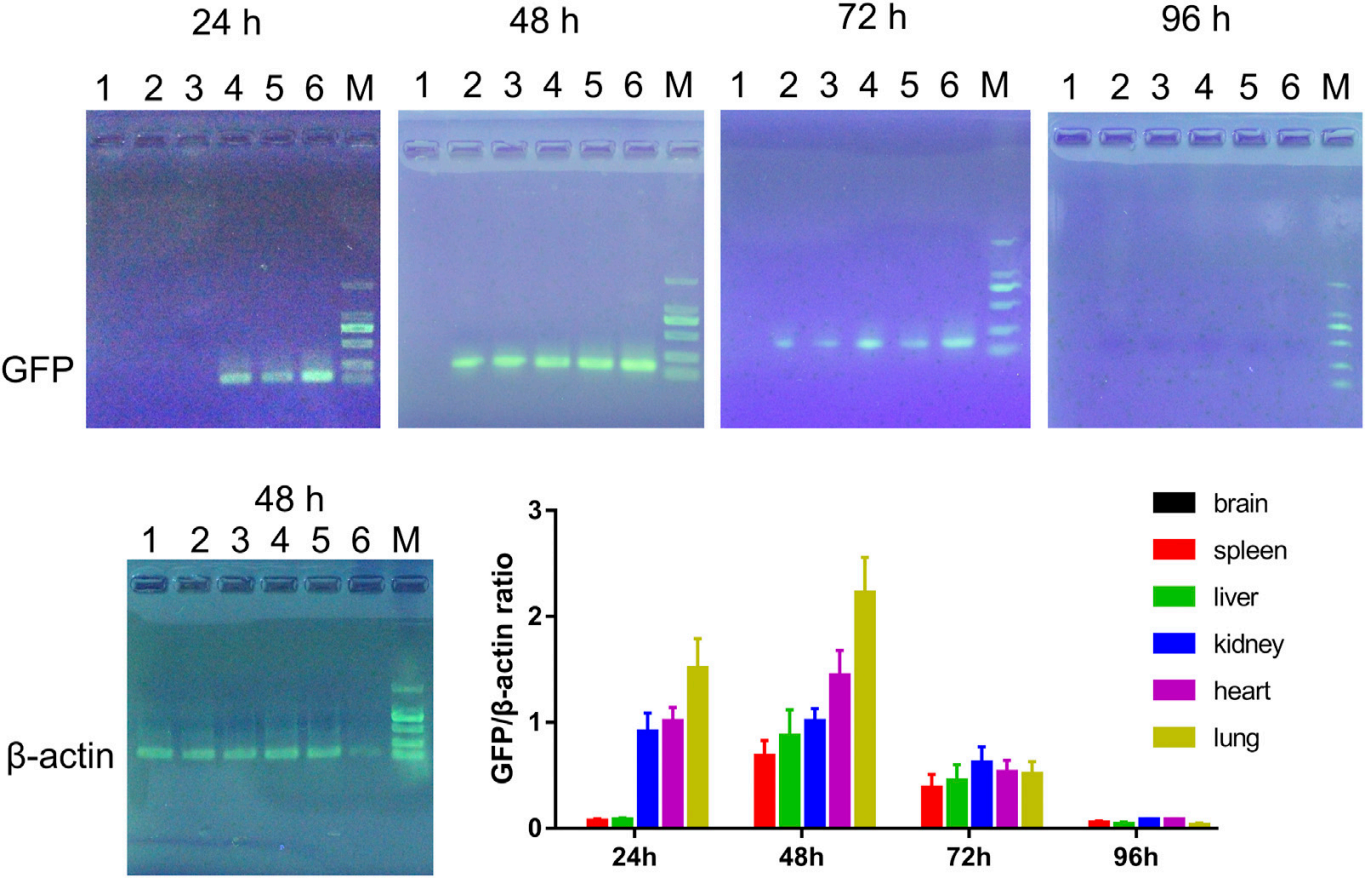
Representative electrophoresis images of RT-PCR products and the quantitative data. 1: Brain; 2: Spleen; 3: Liver; 4: Kidney; 5: Heart; 6: Lung; M: DL2000 marker. *n* = 3.

To assess the distribution of double gene modified hucMSCs *in vivo*, the mice were injected with LV-scFv-sTRAIL infected hucMSCs. The presence of exogenous double gene modified hucMSCs in the brain, spleen, liver, kidney, heart and lung were revealed by RT-PCR analysis for GFP. As shown in Figure 8, 24 h after injection, hucMSCs were present in the kidney, heart and lung, but not brain, spleen and liver. 48 h after injection, hucMSCs could be detected in the spleen, liver, kidney, heart and lung, but not brain. After 72 h, the number of exogenous hucMSCs in each organ gradually decreased and almost could not be detected after 96 h.

## Discussion

The success of targeted anti-tumor therapy largely depends on an effective targeted drug (gene) delivery system (Zhou et al., 2018). In this regard, hucMSCs exhibited significant tumor migration and could migrate or home to damaged tissues, lesions of the tumor, and metastases after systemic administration (Zhuang et al., 2021). Antibody-mediated targeted therapy is a novel molecular targeting therapy, which can greatly improve the targeting efficiency of genetically modified hucMSC (Ma et al., 2019). In this study, we showed that LV-scFv-sTRAIL elevated the tropism capacity of hucMSCs towards U87G. That indicated that the scFv increased the targeting of hucMSCs to U87G. Huyan (Huyan et al., 2017) found that in the presence of 10 μg/ml anti-HAAH-C mAb, the cytotoxicity of NK cells against hepatoma cell HepG2, cervical cancer cells HeLa, and breast cancer cells MCF-7 increased significantly. Furthermore, research has reported that anti-HAAH mAb (PAN-622) was used to prepare an imaging agent and radioimmunotherapy agent for the detection and treatment of metastatic breast cancer (Revskaya et al., 2017).

The scFv-sTRAIL fusion protein, which can be fused by tumor selective scFv and sTRAIL, can target and induce apoptosis of tumor cells, and it is expected to become a new candidate drug for tumor treatment (Muthu et al., 2020). Liu et al. (2021) demonstrated that sTRAIL gene-modified adipose-derived stem cells obtained tumor-restraining effect on hepatocellular carcinoma cells. The sTRAIL has tumor-suppressive activity on circulating CD44^+^ cells in patients with non-small cell lung cancer (Sun et al., 2020). The scFv425:sTRAIL fusion protein, comprises the EGFR blocking antibody fragment scFv425 and sTRAIL, could induce apoptosis of EGFR-positive tumor cells (Bremer et al., 2005). Nevertheless, the effect of the scFv-sTRAIL fusion protein on glioma development is still unclear. In this study, the U87G proliferation was significantly inhibited by hucMSCs infected with LV-scFv-sTRAIL. The apoptosis of U87G was induced by hucMSCs infected with LV-scFv-sTRAIL in co-culture method. In the *in-vivo* experiments, the weight and volume of glioma tumor were significantly inhibited by hucMSCs infected with LV-scFv-sTRAIL. The cell apoptosis of glioma tumor was obviously induced by hucMSCs infected with LV-scFv-sTRAIL. The double gene modification also enhanced the tropism ability of hucMSCs towards the xenograft tumor. Thus, the LV-scFv-sTRAIL infection enhanced the capacity of hucMSCs to target and kill gliomas cells *in vitro* and *in vivo*.

The BBB is a unique border separates brain parenchyma from the bloodstream. As reported, about 98% of prospective medications for brain disorders fail to penetrate BBB (Yarygin et al., 2021). Whether MSCs can cross the BBB is crucial for the clinical application of glioma treatment. Previous reports have shown that there was only 0.0005% MSCs cross the BBB in a traumatic brain injury rat model after intravenous injection (Harting et al., 2009). In another study, most of the injected hucMSCs were found to be distributed in the lung, heart, and liver, but were not detected in the brain at any of the time points after a single intravenous injection in a Alzheimer’s disease mouse model (Park et al., 2016). In this study, we detected the distribution of double gene modified hucMSCs *in vivo* by RT-PCR according to the methods of a previous study (Cafforio et al., 2017). The mice were injected with LV-scFv-sTRAIL infected hucMSCs *via* the tail vein. The organs of mice were collected at different time points for RT-PCR detection. The results showed that LV-scFv-sTRAIL infected huMSCs could be detected in the spleen, liver, kidney, heart and lung, but not in brain. This distribution pattern was in line with a previous study (Park et al., 2016). Since the main purpose of this study was to investigate whether the double gene-modified hucMSCs could inhibit tumor growth *in vivo*, a mouse xenograft glioma model was used. The orthotopic glioma model was not used to investigate whether the double gene modified hucMSCs could cross the BBB. This is the main limitation of this study. It had been reported in the literature that the MSCs could migrated through the BBB for the treatment of brain diseases (Yarygin et al., 2021). But we did not detect the presence of exogenous hucMSCs in the present study, which is in line with expectations. The main reasons might be as follows: The subjects in the previous studies, in which the MSCs could cross the BBB, always had brain disease, such as glioma (Pacioni et al., 2017; Abdi et al., 2018), traumatic brain injury (Darkazalli et al., 2017), ischemic stroke (Do et al., 2021; Yarygin et al., 2021) or encephalitis (Bian et al., 2017). In these cases, the BBB was damaged, and lots of inflammatory mediators or chemokines released from inflammatory or tumor lesions in the brain became a decisive factor in promoting the recruitment and migration of therapeutic MSCs through BBB into infarct areas (Huang et al., 2018; Al-Kharboosh et al., 2020). However, in this study, the subjects we used to study the distribution of hucMSCs *in vivo* were normal healthy mice, which lacked the above-mentioned inflammatory mediators or chemokines to induce and recruit transfused exogenous hucMSCs through BBB. In our next research, we will construct an orthotopic glioma mouse model in the brain. Using the model, we will focus on whether these double gene-modified hucMSCs can cross the BBB. This is very important for the clinical application of this double-gene modified hucMSCs in the treatment of glioma.

In summary, the present study demonstrated that the hucMSCs expressing scFv-sTRAIL fusion protein exhibited significant tropism toward glioma, and inhibited the growth of glioma *in vitro* and *in vivo*. These findings shed light on a potential therapy for glioma treatment.

## Data Availability

The raw data supporting the conclusions of this article will be made available by the authors, without undue reservation.
